# Fourteenth Annual Meeting of the American Medical Association

**Published:** 1863-06

**Authors:** 


### EDITORIAL DEPARTMENT.

### FOURTEENTH ANNUAL MEETING OF THE AMERICAN MEDICAL ASSOCIATION.

From the Chicago Tribune.

### TUESDAY MORNING SESSION.

The meeting of the American Medical Association commenced its sessions this morning at Bryan Hall, Chicago, at 11 o'clock. The attendance was large, and comprised the following gentlemen:

*Vermont*—G N Stiles, Lewis Emmons.

*Massachusetts*—Henry Cutter, Appleton Thorne, Edward Barton, Jas. P Lynde, Ebenezer Stone, P J Kendall, B E Cotting, Joshua Homans, John C Dalton, M D Southwick, E P Abbe, John Green.

*New York*—Henry G Davis, Guido Furman, Alden March, Daniel P Bissell, James S Whaley, Thomas C Brimsmade, J S Sprague, C C F Gay, Edward Hook, E S F Arnold, E W Cherry, W N Blakeman, Howard Townsend, H Nichell, E Tobie, H S Downs, C C Wyckoff, S Eastman, Alf. Underhill, J H Griscom, L B Cotes, Julius Homberger, C W Harvey, J McNaughton, D Holmes.

*Connecticut*—Stephen G Hubbard, L N Beardley, B H Catlin, A W Barrows.

*New Jersey*—Wm Pierson, jr., D M Sayre, John Blain, Isaac S Cramer.

*Delaware*—H F Askew, James Cooper.

*Pennsylvania*—Wilson Jewell, Wm Mayberry, Edward Wallace, B Richardson, John R Thomas, E H Mason, Wm L Richardson, T N Troth.

*Virginia*—J C Hupp.

*Ohio*—W S Battles, J M Taggert, W W Jones, A H Agard, K G Thomas, S O Almy, L N Lawson, W B Davis.

*Indiana*—B S Woodworth, A M Vickery, A J Erwin, A P Ferris, L D Personett, Samuel Ferris, S A Freeman, L D Glazebrook, Jas F Hibbard.

*Michigan*—A B Palmer, E A Eggery, H O Hitchcock, S D Richardson, Lewis Davenport, E W Jenks.

*Illinois*—E L Holmes, Geo K Amerman, Edward Andrews, John Ten Broek, C R Parks, T D Fisher, Geo W Hall, David Prince, E Andrews, N Wright, W O Chamberlain, J P Rouse, E A Steele, A Fisher, M J Johnson, J H Hollister, D Pierson, M F Dewitt, S Wirkerster, J D Rose, Henry Wing, Charles Gorham, S W Noble, Ira Hatch, T F Worrell, T P Haller, H A Johnson, R Spitler, G Pasli, H Noble, D M Tarker, Orrin Smith, A J Craine, T K Edmiston, J J Lake, Hiram Nance, A L Merriam, V C Secord, H W Jones, D D Breagle, T Bevan, H N Hurlbut, S Eargle, T D Fitch.

*Wisconsin*—Charles L Stoddert, H Adams, Harmon Van Duson, E S Carr, George D Wilber, S W Bicknerd.

*Iowa*—J W H Baker, Samuel C Lay, Joseph Sprague, D L McGuggin.

*Kansas*—D W Stormont, C A Logan,

*Tennessee*—W K Boling.

*Army and Navy*—C C Cox, O Simpson, A R Terry, John R Porter, Henry Palmer, M K Taylor, Ralph Isham.

The deliberations of the session were presided over by the first Vice President, Dr. Wilson Jewell, of Pennsylvania, supported by Vice President Dr. A. B. Palmer, of Michigan. Secretaries Drs. S. G. Hubbard, of Connecticut, and H. A. Johnson, of Illinois.

Rev. R. L. Collier invoked the Divine blessing and direction of the meeting.

Dr. N. S. Davis welcomed the members to the City of Chicago. They had heretofore met in cities at the East, where years of improvement had wrought more perfection than in Chicago, but here were all things pertaining to those cities, in their incipiency. He could point to our great granaries, our great commercial interests, our schools, our universities, and our every element of civilization, where recently the Indian and untutored life was rife, with pride. New as Chicago is, she is not behind the age. The same patriotism, friendship, and harmony of interests exist, and he again welcomed them to all the hospitality which Chicago could offer.

The Committee of Arrangements then made their report, through the Chairman, Dr. N. S. Davis, explaining why no regular meeting of the Association has been held since 1860—which may be summed up in a very few words—the unsettled state of the country, owing to the rebellion, and the indisposition of the various railroads to convey members at reduced rates—the latter a difficulty which was happily removed by the arrangements entered into for the Ship Canal Convention. The details of the work during the session were then fully explained, as noted in another place, as well as for the entertainments provided for each evening. The report was accepted and adopted.

At the close of his address he read a statement of the reasons for the postponement of the regular Annual Meeting of the Association, and the unanimity of sentiment in favor of calling the present meeting in Chicago. This communication embraced, in brief, a history of the Association, and of its past deliberations to the present time.

The Chairman of the Committee of Arrangements then announced the following

PROGRAMME OF EXERCISES, &C.

General Session, from 9 o'clock A. M. to 1 o'clock P. M. Afternoon Session, in Sections, from 3 o'clock P. M. to 6 P, M., in rooms, as follows:

Section on Practical Medicine and Obstetrics, in Bryan Hall, No. 2.

Section on Anatomy and Physiology, in Methodist Church Block, 3d floor.

Section on Chemistry and Materia Medica, in the Methodist Church Block, 4th floor.

Section on Meteorology, Medical Topography, Epidemic Diseases, Medical Jurisprudence and Hygiene, in Methodist Church Block, 3d floor.

Seats will be reserved in the gallery for ladies during all general sessions.

A recess of ten minutes was announced by the Chair, to enable delegates to select from their number nominating delegates. After which the retiring acting President Wilson Jewell, of Pennsylvania, 1st Vice President, delivered his valedictory, which was an able, patriotic and scientific address, and which, under other circumstances, we should publish entire.

On motion of Dr. Sprague, of New York, the thanks of the Convention were tendered for the address, and a copy requested for publication.

The Chairman of the Committee of Arrangements presented the name of the following physicians, who were duly elected members of the Association:

Walter Hay, Thomas Bevan, John McAlister, John Bartlett, M. O. Hey. cock, N. P. Peterson, R. C. Hamill, H. N. Hurlbut, V. L. Hurlbut and H W. Jones, of Chicago; S. E. Gardner, Vermont, Ill.; H. Durbam, LaSalle' Ill.; Silas Earle, Onargo, Ill.; W. W. Sedgwick, Sandwich, —; S. W. Bickwell, Beloit, Wis.; E. W. Jerks, Mich.; Daniel B. Brengle, Winchester, Ill.; V. C. Secord, Galva, J. B. Samuel, Carrolton, James S. King, Lemont, and D. F. Crouse, Mt. Carrol, Ill.

AFTERNOON SESSION.

On motion, meeting adjourned to 3 o'clock P. M,

Dr. Haswell of Delaware, read the Treasurer's Report—recommending that only papers of greatest importance should be published in the transactions, owing to increased expense of printing and publishing. Expended since last meeting, $2,559.86. Balance on hand $504.21. He also read the report of the Committee of Publication, which was accepted.

Dr. Driscomb of New York, read an account of a remarkable case of diarrhœa, adiposa, which has no parallel in medical records.

Dr. D. L. McGugin of Iowa, from the Committee on Prize Essays, reported an Essay entitled, "An inquiry into the Physiological and Medicinal properties of the Veratrum Viride, together with some Physiological and Chemical observations upon the Alkaloid Veratria obtained from this and other species," by Samuel R. Percy, M. D., Professor of Materia Medica and Therapeutics, New York Medical College.

After commendatory remarks from several members, the Essay was decided to be worthy of publication in the Transactions, and was entitled to the prize—$100, and it was thus received and disposed of.

The Committee on Nominations reported that they have nominated the following, who were subsequently elected as the

OFFICERS FOR THE ENSUING YEAR.

*President*—Dr. Alden March, of New York.

*Vice Presidents*—Dr. James Cooper of Delaware, David Prince of Illinois, Dr. C. C. Cox of Maryland, and Dr. E. S. Carr of Wisconsin.

*Treasurer*—Dr. Cooper Wistar of Philadelphia.

The election of Secretaries was deferred till the place of the next meeting was decided upon.

The newly elected officers were conducted to their respective positions.

Reports were called for from Committees. Dr. D. L. McGugin of Iowa, Dr. C. C. Cox of Maryland, made verbal reports, and were continued on committees. Dr. Davis read a communication from Dr. Squibb of New York—of Committee on Practical Workings of the United States Law relating to the Inspection of Druga and Medicines. He was unable to report—continued. Dr. A. K. Gardner's paper "On the Use and Abuse of Pessaries," was presented, and the reading postponed till Monday morning. Dr. C. C. Cox of Maryland, asked and was granted a few weeks to complete his report on "American Medical Nicrology," also continued on same committee for another year.

A letter from H. J. Bowditch of Boston, was read, announcing the receipt of $356 toward the Hnnter Memorial, with a list of officers and agents, and on motion the Committee were instructed to forward the amount of funds on hand, and to close the account.

On motion of Dr. Lawson of Ohio, it was

*Resolved,* That a committee of one from each State represented in this Association be appointed to inquire into a recent order issued by the Surgeon General of the United States Army, in which the further supply of calomel and antimony is prohibited, and to report at as early a period as convenient, during the session of the Association. Adopted.

Dr. Arnold of New York, submitted to the Convention his pamphlet on "Medical Provision for Railroads as a Humanitarian Measure, as well as a source of economy to the Companies." Copies of this work (which embraces an account of all that has been accomplished by the profession to the present time,) were presented to the members, with the request that after being read, the subject be brought up for discussion at some period of this session.

Dr. C. C. Cox of Maryland, offered the following resolutions, with an appropriate introduction, which, after endorsing remarks from several members, were adopted unanimously:

*Resolved,* That a committee of five be appointed by the Chair to draft a memorial to Congress, asking the enactment of a law by which surgeons in the service of the United States army may be accorded relative rank in the same.

*Resolved,* That each medical gentleman present be urgently invited to use every proper influence with the member of Congress from his respective district, to urge the passage of a law, at the coming session of Congress, favorable to this object.

The Association adjourned to 9 A. M., Wednesday.

To be concluded in July number.

---

